# The angiotensin II type 2 receptor antagonists, PD123,319 ((*S*-( +)-1-[(4-(dimethylamino)-3-methylphenyl)methyl]-5-(diphenylacetyl)-4,5,6,7-tetrahydro-1H-imidazo[4,5-c]pyridine-6-carboxylic acid), EMA300 (5-(2,2-diphenylacetyl)-4-[(4-methoxy-3-methylphenyl)methyl]-1,4,6,7-tetrahydroimidazo[4,5-c]pyridine-6-carboxylic acid) and EMA401 ((3*S*)-5-(benzyloxy)-2-(2,2-diphenylacetyl)-6-methoxy-1,2,3,4-tetrahydroisoquinoline-3-carboxylic acid), evoke pain relief in a varicella zoster virus-induced rat model of neuropathic pain

**DOI:** 10.1007/s10787-025-01650-z

**Published:** 2025-02-20

**Authors:** V. Das, A. L. Lam, M. T. Smith

**Affiliations:** Centre for Integrated Preclinical Drug Development (CIPDD), School of Biomedical Sciences, Faculty of Medicine, The University of Queensland, St Lucia Campus, Brisbane, QLD 4072 Australia

**Keywords:** Varicella zoster virus (VZV), Neuropathic pain, PD123,319, EMA300, EMA401, AT_2_ receptor antagonist

## Abstract

Post-herpetic neuralgia (PHN) is a type of neuropathic (nerve) pain that persists for more than 3 months after crusting of the last shingles lesion. It is difficult to relieve with analgesic/adjuvant medications, and so novel analgesics are needed. Our aim was to use a rat model of varicella zoster virus (VZV)-induced neuropathic pain to assess the pain relief efficacy of several small molecule angiotensin II type 2 (AT_2_) receptor antagonists (PD123,319, EMA300, and EMA401) relative to clinically used analgesic/adjuvant agents from four different pharmacological classes. Male Wistar rats received a unilateral intraplantar injection of VZV-infected MRC-5 cells (2 × 10^4^ infected cells) and paw withdrawal thresholds (PWTs) in the ipsilateral hindpaws were assessed using von Frey filaments. Animals with PWTs ≤ 8 g received single doses of PD123,319 (0.03–3 mg/kg), EMA300 (0.3–5 mg/kg), EMA401 (0.03–1 mg/kg), gabapentin (10–60 mg/kg), amitriptyline (5–30 mg/kg), morphine (0.1–3 mg/kg), meloxicam (5–20 mg/kg) or vehicle and PWT versus time curves were generated. Single doses of PD123,319, EMA300, EMA401, gabapentin and morphine-evoked dose-dependent anti-allodynia in the hindpaws of VZV-rats. The mean (95% confidence intervals) ED_50_s were 0.57 (0.04–1.7), 2.5 (1.0–3.7) and 0.41 (0.12–0.87) mg/kg for PD123,319, EMA300, and EMA401, respectively. The ED_50_s for gabapentin and morphine were 39.9 (25.1–64.8) and 0.04 (0.16–2.09) mg/kg, respectively. In conclusion, the anti-allodynic efficacy of EMA401 in a VZV-rat model of neuropathic pain is aligned with its analgesic efficacy in a Phase 2a clinical trial in patients with PHN. This model has utility for anti-allodynic efficacy assessment of novel AT_2_ receptor antagonists from drug discovery.

**Supplementary Information:**

The online version contains supplementary material available at 10.1007/s10787-025-01650-z.

## Introduction

Post-herpetic neuralgia (PHN), a type of peripheral neuropathic (nerve) pain, is often intractable to relief with clinically used analgesic/adjuvant agents (Opstelten et al. 2010). Thus, there is a large unmet medical need for novel analgesics to improve relief of PHN.

To date, a huge research effort has been directed at identifying novel targets for use in analgesic discovery programs (Smith 2022). One such target is the angiotensin II type 2 (AT_2_) receptor (Smith 2022; Smith and Muralidharan 2015; Shepherd et al. 2023). Previously, we showed that several small molecule AT_2_ receptor antagonists (PD123,319, EMA300, and EMA400) with > 1000-fold selectivity over the angiotensin II type 1 (AT_1_) receptor, evoked pain relief (anti-allodynia) in the chronic constriction injury (CCI)-induced rodent model of neuropathic pain (Smith et al. 2013a, b). Anti-allodynia was abolished in AT_2_ receptor knockout CCI-mice affirming the AT_2_ receptor as the drug target (Smith et al. 2013a). In the CCI-rat (Smith et al. 2013a) and in a rat model of mixed inflammatory and neuropathic pain (Muralidharan et al. 2014), single doses of EMA300 and PD123,319, respectively, at the time of peak effect, attenuated otherwise augmented Ang II expression levels in the ipsilateral lumbar DRGs (Smith et al. 2013a). In addition, at the time of peak pain relief, ipsilateral lumbar DRG expression levels of phospho-p38 mitogen-activated protein kinase (pp-38 MAPK) and phospho-extracellular signal-related kinase 1/2 (pERK1/2) were attenuated to match the corresponding sham-levels (Smith et al. 2013a). These findings are aligned with work in cultured rat DRG neurons incubated with Ang II (endogenous AT_2_ receptor agonist) or the small molecule AT_2_ receptor agonist, compound 21, which showed increased formation of pp-38 MAPK and pERK1/2 that was attenuated by co-incubation of cultured neurons with EMA401 (Anand et al. 2015).

Encouragingly, the pain relief data evoked by AT_2_ receptor antagonists in rodent pain (Smith 2022; Shepherd et al. 2023), translated into a successful Phase 2a clinical trial of EMA401 in patients with PHN (Rice et al. 2014). However, two subsequent Phase 2b clinical trials of EMA401 in patients with PHN and with painful diabetic neuropathy were terminated early due to unexpected hepatotoxicity in a long-term (39 weeks) toxicity study that was undertaken concurrently in cynomolgus monkeys (Rice et al. 2021). Nevertheless, as the AT_2_ receptor is clinically validated for alleviating neuropathic pain, other small molecule AT_2_ receptor antagonists are being progressed into clinical development based upon the hypothesis that EMA401-induced hepatotoxicity in monkeys is molecule specific (Shepherd et al. 2023).

To facilitate discovery of novel AT_2_ receptor antagonists for relief of PHN, we established a VZV-induced rat model of neuropathic pain based upon previous work by others (Dalziel et al. 2004; Kinchington and Goins 2011; Guedon et al. 2015). We validated this model by showing that the AT_2_ receptor antagonists, PD123,319, EMA300 and EMA401, evoked dose-dependent pain relief and the data so generated are described herein.

## Methods

**Fig. 1:**
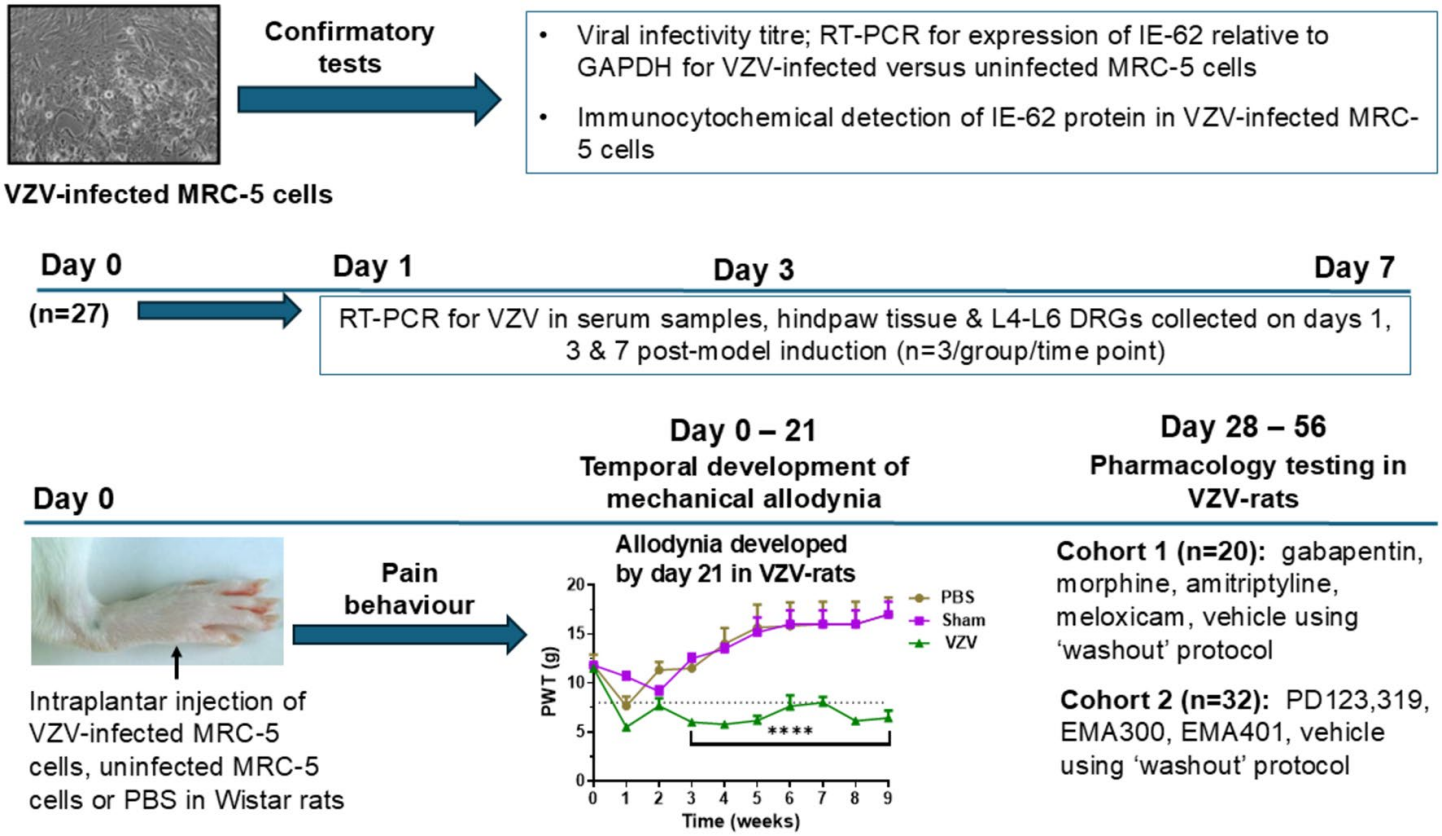
Schematic overview of the experiments undertaken to establish the VZV-induced rat model of neuropathic pain and the use of this model to define the comparative anti-allodynic effects of single bolus doses of three small molecule AT_2_ receptor antagonists (PD123,319, EMA300, and EMA401) relative to single bolus doses of gabapentin, morphine and vehicle

An overview of the experimental paradigm used herein to induce a rat model of VZV-induced neuropathic pain which was then used to assess the pain relief efficacy of single bolus doses of several small molecule AT_2_ receptor antagonists relative to that for single bolus doses of gabapentin, morphine, amitriptyline and meloxicam is shown in Fig. 1.

### Cell culture

MRC-5 cells (CCL-171TM) and the Ellen strain of VZV (VR-1367TM) were from the American Type Culture Collection (ATCC; Manassas, VA, USA). Minimum essential medium (MEM), foetal bovine serum (FBS), 100 U penicillin–streptomycin, glutamax, sodium pyruvate, 0.25% trypsin–0.53 mM ethylenediaminetetraacetic acid (EDTA), and Dulbecco’s phosphate buffer saline (PBS) were from Invitrogen (VIC, Australia).

### Propagation of VZV-infected MRC-5 cells

MRC-5 cells were cultured (5% CO_2_ at 37 °C) to ~ 80% confluence in minimum essential medium (MEM) containing 10% FBS, Glutamax (2 mM), sodium pyruvate (1 mM) and 100 U penicillin–streptomycin (Lambert and Pirt 1976; Uemura et al. 2021). MRC-5 cells were infected with the Ellen strain of VZV and propagated according to the methods described in the Supplementary Methods (see Figure S1). VZV IE-62 62 (immediate-early 62) protein was detected in VZV-infected, but not uninfected, MRC-5 cells using immunocytochemistry (Figure S2).

### Test compounds and reagents

**Fig. 2:**
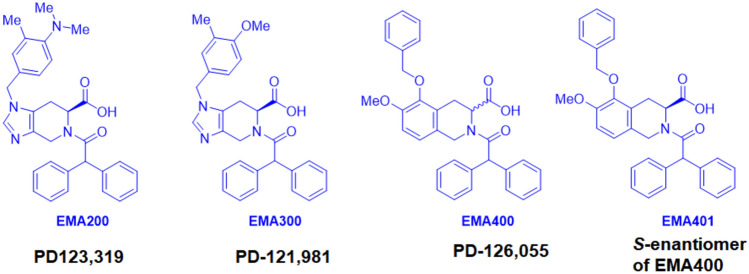
Chemical structures of the small molecule AT_2_ receptor antagonists, PD123,319 (also referred to as EMA200), its structural analogue, EMA300 (also referred to as PD121,981), EMA400 (also referred to as PD126,055), and EMA401 ([*S*]-enantiomer of EMA400) (from Smith 2022)

Gabapentin was from Dr. Ben Ross, School of Pharmacy, The University of Queensland, Australia. Morphine sulphate ampoules were from Hospira Australia Pty Ltd (Melbourne, Vic, Australia). Meloxicam sodium salt hydrate and amitriptyline hydrochloride were from Sigma Aldrich (Sydney, NSW, Australia). PD123,319 ((*S*-( +)-1-[(4-(dimethylamino)-3-methylphenyl)methyl]-5-(diphenylacetyl)-4,5,6,7-tetrahydro-1H-imidazo[4,5-c]pyridine-6-carboxylic acid as the ditrifluoroacetate salt), also known as EMA200, was from Tocris Biosciences (Bristol, UK). EMA300 (5-(2,2-diphenylacetyl)-4-[(4-methoxy-3-methylphenyl)methyl]-1,4,6,7-tetrahydroimidazo[4,5-c]pyridine-6-carboxylic acid) and EMA401 ((3*S*)-5-(benzyloxy)-2-(2,2-diphenylacetyl)-6-methoxy-1,2,3,4-tetrahydroisoquinoline-3-carboxylic acid) as the sodium salts were from Glycosyn^irl^ (Lower Hutt, New Zealand) and were a kind gift from Spinifex Pharmaceuticals Pty Ltd (Melbourne, Australia). EMA300 is a structural analogue of EMA200 and EMA401 is a member of the tetrahydroisoquinoline class of AT_2_ receptor antagonists (Fig. 2). Isoflurane was from Abbott Australia, Pty. Ltd. (Sydney, NSW, Australia). Pentobarbitone sodium (Lethabarb®) was from Virbac, Australia Pty. Ltd. (Crookwell, NSW, Australia). Medical grade oxygen was from Core Gas (Brisbane, QLD, Australia).

### Animals

Animal ethics approval (CIPDD/TETRAQ/152/10/ARC) was from The University of Queensland and experiments adhered to the Australian Code of Practice for the Care and Use of Animals for Scientific Purposes (NHMRC 2013). Male Wistar rats were from the Animal Resources Centre (Perth, Australia) or The University of Queensland Biological Resources. Animals were housed in pairs in a temperature-controlled facility (21 ± 2 °C) with a 12/12-h light–dark cycle, and experimentation was conducted during the light phase. Standard rodent chow and water were available ad libitum. Kim-wipes and rodent hutches constructed from red Perspex were used for environmental enrichment.

### Establishment of VZV-induced rat model of neuropathic pain

Two cohorts of male Wistar rats (200–300 g) were anesthetised with 3% isoflurane delivered in oxygen to facilitate unilateral intraplantar (i.pl.) injection (50 μL) of 2 × 10^4^ MRC-5 cells infected with the Ellen strain of VZV. In both cohorts, experimentation was conducted during the light phase. Initially for Cohort 1, baseline paw withdrawal thresholds (PWTs) were assessed in the hindpaws using calibrated von Frey filaments (range 2–20 g) (Stoelting Co., Wood Dale, IL, USA). Next, rats were randomised to receive a single unilateral i.pl. injection (50 μL) of one of the following treatments: (i) phosphate-buffered saline (pH = 7.4, 1 mM; *n* = 4); control group; (ii) uninfected MRC-5 cells (7 × 10^5^ cells; *n* = 4); sham group; (iii) MRC-5 cells infected with the Ellen strain of VZV (2 × 10^4^ infected cells, *n* = 20). Rats were kept warm and monitored closely in the post-inoculation period. Thereafter, rats were housed in pairs in individually ventilated cages in the animal holding facility. Whilst anaesthetised with 3% isoflurane delivered in oxygen, Cohort 2 rats (*n* = 32) received a unilateral i.pl. injection (50 μL) of 2 × 10^4^ VZV (Ellen strain)-infected MRC-5 cells. Both cohorts of rats were kept warm and monitored during anaesthetic recovery. Post-recovery, they were housed in pairs in individually ventilated cages in the animal holding facility.

#### Assessment of mechanical allodynia in the hindpaws

Von Frey filaments (range 2–20 g) (Stoelting Co., Wood Dale, IL, USA) were used to define the time course for the development of mechanical allodynia in the bilateral hindpaws of these animals. Briefly, baseline von Frey paw withdrawal thresholds (PWTs) were measured for the ipsilateral hindpaws at once-weekly intervals for the 8-week study period. On each testing occasion, rats were acclimatised for ~ 20–25 min in wire mesh cages (20 × 20 × 20 cm) and baseline PWTs were determined in triplicate according to the “up down method”, with approximately 5 min between successive measurements. Specifically, von Frey filaments were used to identify the lowest mechanical threshold to evoke a brisk hindpaw withdrawal reflex starting with the 6 g filament and then selecting filaments in 2 g increments up or down depending upon the response. The absence of a response after ~ 3 s prompted application of the next filament of increasing force. A score of 20 g was given to animals that did not respond to any of the filaments (Shenoy et al. 2018). Mechanical allodynia was fully developed in the hindpaws when von Frey PWTs were ≤ 8 g.

### Detection of VZV by RT-PCR

An additional group of male Wistar rats (200–230 g; *n* = 27) was used to assess detection of VZV by RT-PCR (reverse transcription-polymerase chain reaction) in this rat model. Briefly, rats received a unilateral i.pl. injection (50 μL) of (i) MRC-5 cells infected with the Ellen strain of VZV (2 × 10^4^ infected cells), (ii) uninfected MRC-5 cells (7 × 10^5^ cells), sham group, or (iii) PBS (pH = 7.4, 1 mM), control group (*n* = 9/group). Rats (*n* = 3 at each of 24 h, 48 h and 7 days post-i.pl. injection) from each of the groups were euthanised with an overdose (100 mg/kg i.p.) of sodium pentobarbitone (Lethabarb®).

#### Serum sample collection

Prior to sample collection, the work area and instruments were cleaned with a 70% ethanol solution followed by a rinse with DNase/RNase free water (UltraPure water). Blood samples (1 mL) were collected via cardiac puncture using a sterile syringe and 25G 5/8 (0.5 ×16 mm) needle into DNase/RNase-Free tubes. Collected blood samples were allowed to clot (20–30 min at room temperature) and then centrifuged at 2500*g* for 10 min at room temperature. Thereafter, the supernatants were transferred into DNase/RNase-Free tubes. Sterile transport swabs (CAT. No. 124C, Copan Diagnostics INC, USA) were soaked with each serum sample and stored in the corresponding swab tubes and refrigerated (4–8 °C) until transported to a commercial pathology laboratory (QML Pathology™; Brisbane, Australia) for RT-PCR analysis using a proprietary standardised QML Pathology™ protocol. All samples were analysed within 3–4 days from the time of collection.

#### Ipsilateral hindpaw swab collection

The ipsilateral (injected) hindpaw skin was cleaned with 70% ethanol solution followed by DNase/RNase-Free water (Invitrogen (Melbourne, VIC, Australia). Thereafter, sterile scissors were used to incise the glabrous skin and expose the subcutaneous tissue. A sterile transport swab was used to swab each hindpaw tissue. The swabs were stored in pre-labelled swab tubes and refrigerated (4–8 °C) until transported to QML Pathology for RT-PCR analysis using a proprietary standardised QML Pathology protocol. All samples were analysed within 3–4 days from time of collection.

#### Dorsal root ganglia (DRGs) collection for RT-PCR

Lumbar (4–6) DRGs were collected from both the ipsilateral and contralateral sides of the same rats used for blood sample collection. DRGs were placed into DNase/RNase-Free water (200 µL) in DNase/RNase-Free tubes and fresh tissue homogenised on ice (VCX130, Sonics vibracell, Sonics & Material INC, Newtown, USA) to form a uniform homogenate (ipsilateral and contralateral DRGs combined). Thereafter, sterile swabs were soaked in the DRG homogenate and placed in the corresponding swab tubes and stored refrigerated (4–8 °C) until transport to QML pathology for RT-PCR analysis using a proprietary standardised QML Pathology protocol. All samples were analysed within 3–4 days from time of collection.

### VZV-rat model of neuropathic pain: pharmacological characterisation

Two cohorts of male Wistar rats (*n* = 20/cohort 1; *n* = 32/cohort 2) were used for this pharmacological characterisation study. Rats with fully developed mechanical allodynia (PWTs ≤ g) in the ipsilateral hindpaws following unilateral i.pl. injection of 2 × 10^4^ VZV-infected MRC-5 cells were used to pharmacologically characterise this rat model of PHN.

**Cohort 1**

#### Gabapentin and morphine

VZV-rats with fully developed mechanical allodynia in the ipsilateral hindpaws at days 21–35 following unilateral i.pl. injection of VZV-infected MRC-5 cells received single subcutaneous (s.c.) doses of gabapentin (10, 30, 60 mg/kg; *n* = 6/group), morphine (0.1, 0.5, 3 mg/kg; *n* = 6/group) or vehicle (*n* = 8). Dosing solutions were masked and administered in a randomised manner by a different laboratory member from the tester (VD) to ensure tester blinding. Dosing was done according to a ‘washout’ protocol such that each rat received up to five single doses with at least 2 days of ‘washout’ between successive doses.

#### Meloxicam and amitriptyline

VZV-rats with fully developed mechanical allodynia in the ipsilateral hindpaws at days 21–35 following unilateral i.pl. injection of VZV-infected MRC-5 cells received single intraperitoneal (i.p.) doses of meloxicam (5.0, 10, 20 mg/kg (*n* = 6/group), amitriptyline (5.0, 10, 30 (*n* = 6/group) or vehicle (*n* = 14). Dosing solutions were masked and administered in a randomised manner by a different laboratory member to ensure tester (VD) blinding. Dosing was performed according to a ‘washout’ protocol such that each rat receiving up to five single doses with at least 2 days of ‘washout’ between successive doses.

**Cohort 2**

#### Small molecule AT_2_ receptor antagonists

Dosing solutions for each of PD123,319, EMA300, EMA401, and vehicle were masked and administered in a randomised manner by a different laboratory member to ensure tester (VD) blinding. VZV-rats received single intravenous (i.v.) doses of PD123,319 at 0.03 (*n* = 8), 0.3 (*n* = 8), 1.0 (*n* = 10) or 3.0 mg/kg (*n* = 10), single i.p. doses of EMA300 at 0.3, 1.0 or 5 mg/kg (*n* = 8/group), single oral (p.o.) doses of EMA401 at 0.03 (*n* = 4), 0.1 (*n* = 9), 0.3 (*n* = 8) or 1.0 mg/kg (*n* = 9), or vehicle (*n* = 20). Each rat received up to five single doses of a test compound or vehicle administered according to a ‘washout’ protocol with at least 2 days of ‘washout’ between successive doses. Ipsilateral hindpaw PWTs were measured pre-dose and at the following post-dosing times, 0.25, 0.5, 0.75, 1.0, 1.25, 1.5 and 2 h as well as at 3 and 4 h for gabapentin.

### Data and statistical analysis

Mean (± SEM) baseline PWT values for VZV-induced rats were plotted against time to produce PWT versus time curves. A two-way ANOVA with Tukey’s multiple comparison test was used to compare temporal changes in PWT values between groups of VZV-rats, sham-rats and rats administered i.pl. PBS. The pharmacological data are presented as mean (± SEM) PWT versus time curves for each dose of each test compound administered. For VZV-rats that received single doses of a test compound or vehicle, PWTs were normalised by subtracting pre-dosing baseline values for each individual rat to obtain ∆PWT values as follows:

$$\Delta {\text{PWT}} = {\text{post - dosing}}\;{\text{PWT}} - {\text{pre - dosing}}\;{\text{baseline}}\;{\text{PWT}}$$

The extent and duration of pain relief (area under the ∆PWT versus time curves (∆PWT AUC values) was estimated using trapezoidal integration for individual rats for each dose of each test compound administered. Dose–response curves were generated by plotting mean (± SEM) ∆PWT AUC values versus log dose for each test compound. The ED_50_ (95% confidence interval) values were estimated using nonlinear regression as implemented in GraphPad Prism™ (v10.1.2). For two-way ANOVA, *F* values are expressed as *F*_df of treatment, time, interaction/residual_. The statistical significance criterion was *P* ≤ 0.05.

## Results

VZV-infection of MRC-5 cells was confirmed by RT-PCR for IE-62 (Table S1). In rats given a unilateral i.pl. injection (50 μL) of VZV-infected MRC-5 cells, but not sham- or control-rats, VZV was detected by RT-PCR in the ipsilateral hindpaw tissue swabs collected at 24 h (3/3) and 48 h (1/3) post-inoculation. However, by day 7, VZV was no longer detectable in the ipsilateral hindpaw tissue swabs from VZV-rats. VZV was not detected by RT-PCR in the serum or the DRG homogenate swabs for any of the time-points evaluated.

### Temporal development of mechanical hypersensitivity in VZV-induced rats

#### General health—body weight

**Cohort 1**

**Fig. 3:**
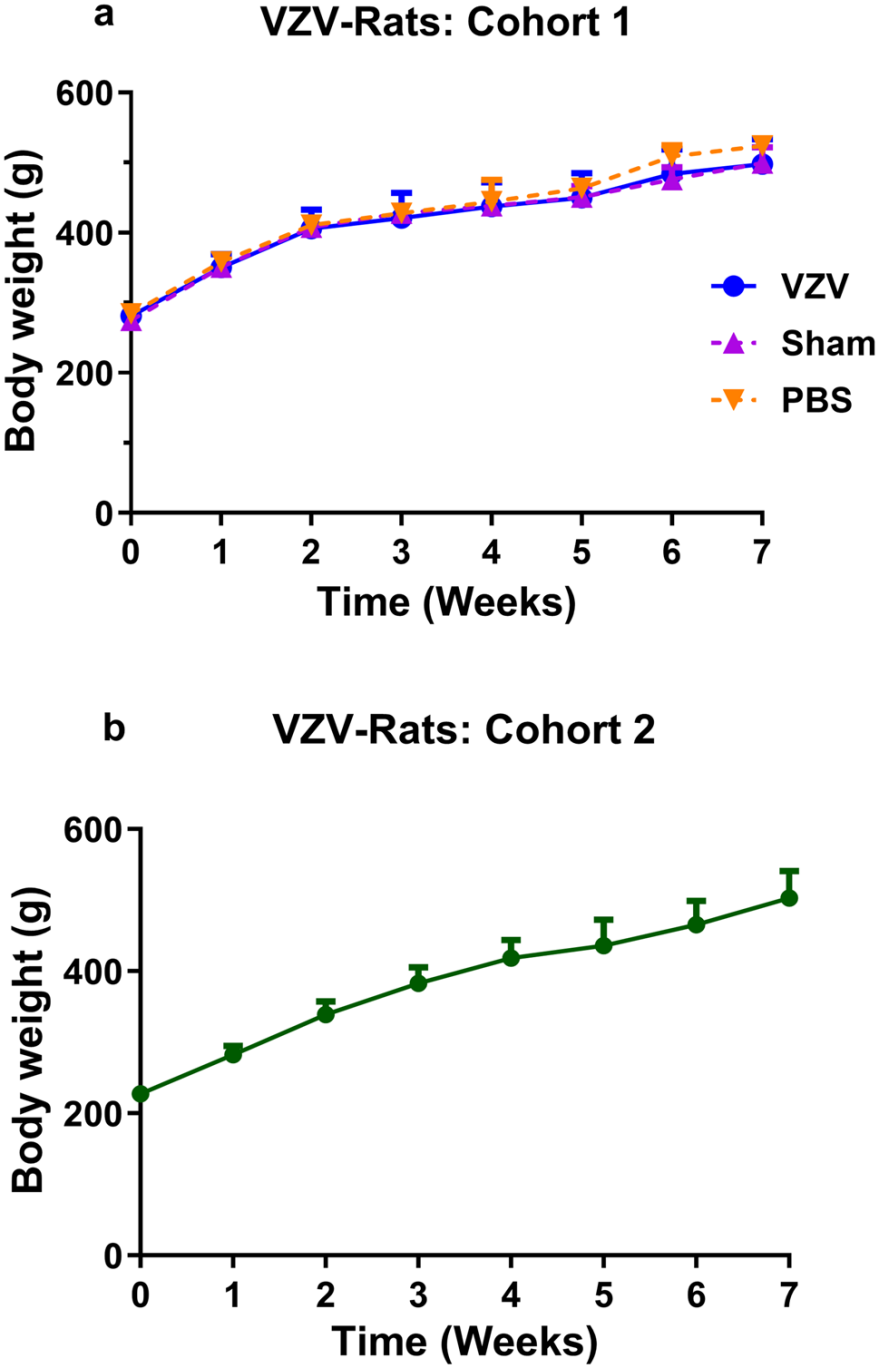
There was a temporal increase in mean (± SEM) body weights of rats throughout the study period irrespective of whether they had received an i.pl. injection of VZV-infected MRC-5 cells, uninfected MRC-5 cells or PBS indicative of good general health of all animals. **a** Mean (± SEM) body weights of Cohort 1 rats administered a unilateral i.pl. injection of MRC-5 cells infected with the Ellen strain of VZV (2 × 10^4^ infected cells; 50 μL) increased significantly throughout the study period in a manner similar to the sham group (7 × 10^5^ uninfected MRC-5 cells; 50 μL) and the control group (PBS 50 μL) (*F*_(2,7,14)_ = 2.614, 122.6, 0.2569, *P* > 0.05, *P* < 0.0001, *P* > 0.05). In addition, the mean (± SEM) body weights of sham- and control-rats did not differ significantly (*P* > 0.05) throughout the study period (two-way ANOVA followed by Tukey’s multiple comparison test. **b** The mean (± SEM) body weights of Cohort 2 VZV-rats administered a unilateral i.pl. injection of 2 × 10^4^ VZV-infected MRC-5 cells increased in a temporal manner throughout the study similar to Cohort 1 VZV-rats

The mean (± SEM) body weights of all rats increased significantly (*F*_(2,7,14)_ = 2.614, 122.6, 0.2569, *P* > 0.05, *P* < 0.0001, *P* > 0.05) in a temporal manner throughout the study period (Fig. 3). Importantly, the mean (± SEM) body weights of rats administered a unilateral i.pl. injection (50 μL) of VZV (Ellen strain) infected MRC-5 cells (2 × 10^4^ infected cells) did not differ significantly (*P* > 0.05) from that of the sham group (uninfected MRC-5 cells; 7 × 10^5^) or the control group (PBS) throughout the 9-week study period (Fig. 3a). Likewise, the mean (± SEM) body weights did not differ significantly (*P* > 0.05) between the sham- and control-groups. Together, these body weight data show that all animal groups maintained good general health throughout the study.

**Cohort 2**

The mean (± SEM) body weight of rats administered a unilateral i.pl. injection (50 µL) of 2 × 10^4^ VZV-infected MRC-5 cells increased steadily throughout the 8-week experimental period (Fig. 3b), mirroring the Cohort 1 (Fig. 3a) VZV-rat body weight data.

### Intraplantar injection of VZV-infected MRC-5 cells induced mechanical allodynia in the rat hindpaws

#### Cohort 1

**Fig. 4:**
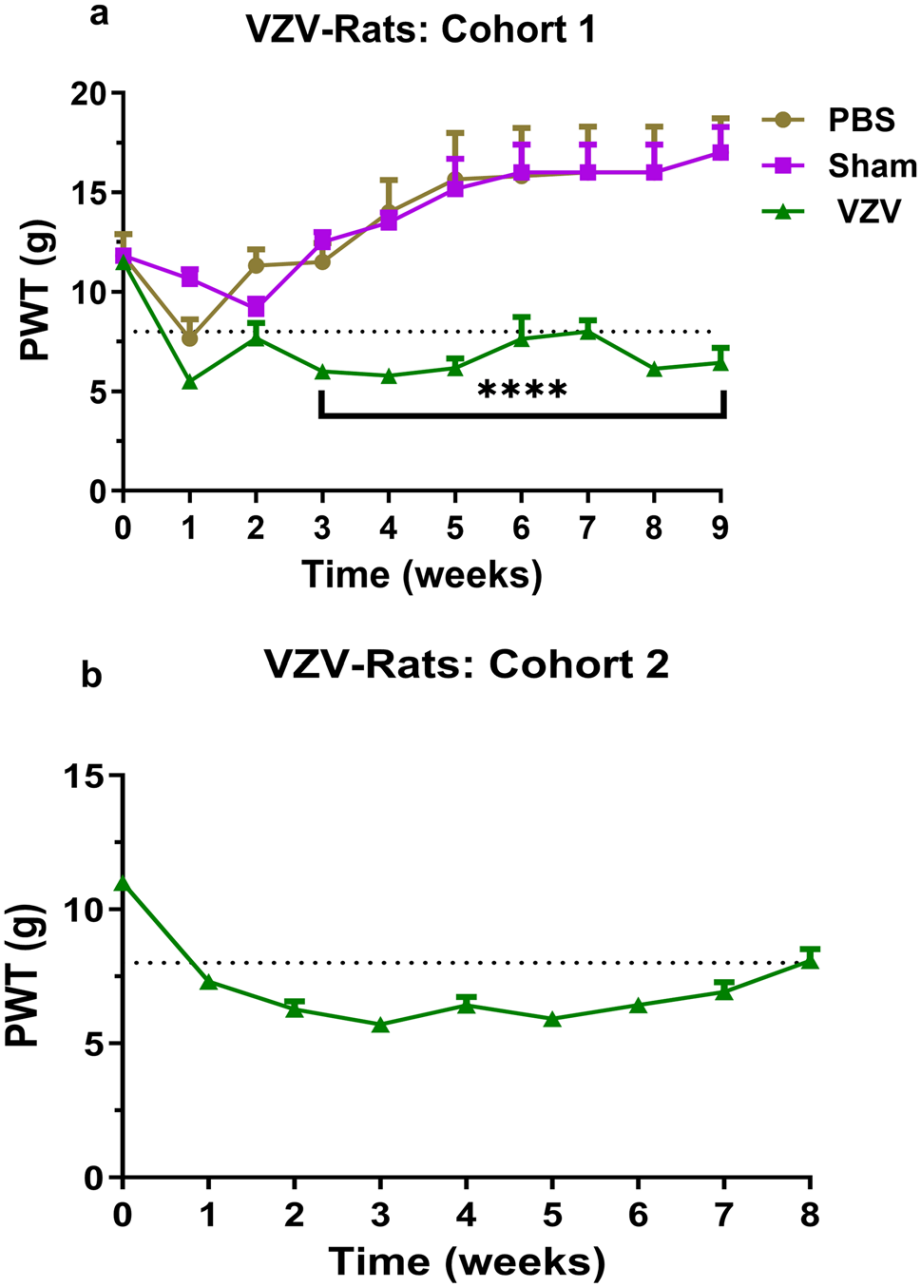
Time-course for the development of mechanical allodynia in the ipsilateral hindpaws in rats following unilateral i.pl. administration of VZV (Ellen strain)-infected MRC-5 cells. **a** Cohort 1 received 2 × 10^4^ VZV-infected MRC-5 cells (50 µL) or uninfected MRC-5 cells (7 × 10^5^; 50 μL) or PBS (50 μL). **b** Cohort 2 received 2 × 10^4^ VZV-infected MRC-5 cells (50 µL). Mechanical allodynia was fully developed (PWTs ≤ 8 g) in the ipsilateral hindpaws by 1-week post-inoculation and maintained for the study duration. **** indicates significant differences (*P* < 0.001) between mean (± SEM) PWT values for VZV-injected rats vis-à-vis sham- and control-rats (repeated-measures two-way analysis of variance (ANOVA) followed by Tukey’s test for repeated-measures)

Following unilateral i.pl. injection of 2 × 10^4^ VZV-infected MRC-5 cells in a volume of 50 μL into the left hindpaw of male rats, there was temporal development of mechanical allodynia in the ipsilateral hindpaws. Hindpaw hypersensitivity was fully developed (PWT ≤ 8 g) by 3 weeks post-inoculation and it was maintained until study completion at 9 weeks post-unilateral i.pl. inoculation (Fig. 4a). Specifically, mean (± SEM) ipsilateral PWTs for VZV-rats were significantly (*P* < 0.05) different (*F*_(1, 9,9/126)=83.71, 3.72, 9.24)_ from the corresponding mean (± SEM) ipsilateral PWTs for sham-rats.

#### Cohort 2

Following unilateral i.pl. injection of 2 × 10^4^ VZV-infected MRC-5 cells into the left hindpaw of rats, there was temporal development of mechanical hypersensitivity in the ipsilateral hindpaws (Fig. 4b), mirroring that for Cohort 1 rats (Fig. 4a). Mechanical allodynia was fully developed (PWT ≤ 8 g) by 1-week post-inoculation in the ipsilateral hindpaws and it persisted until study completion at 8 weeks post-inoculation (Fig. 4b).

### VZV-rat model of neuropathic pain: pharmacological characterisation

The Neuropathic Pain Special Interest Group of the IASP (NeuPSIG) has published evidence-based guidelines for the pharmacological management of neuropathic pain (Finnerup et al. 2015). First-line treatments with a strong recommendation include antidepressants (e.g. tricyclic antidepressants such as amitriptyline), and anticonvulsants (e.g. gabapentin) (Finnerup et al. 2015). Third-line treatments with a weak recommendation include strong opioid analgesics due to their plethora of opioid-related adverse effects including abuse liability, respiratory depression and constipation (Finnerup et al. 2015). Nonsteroidal anti-inflammatory drugs (NSAIDs) are also not recommended due to lack of efficacy (Finnerup et al. 2015). To characterise this VZV-rat model of neuropathic pain, we selected representative clinically used analgesic/adjuvant agents from four classes, viz. gabapentin, amitriptyline, morphine and meloxicam relative to vehicle and several small molecule AT_2_ receptor antagonist investigational agents, viz., PD123,319, EMA300, and EMA401.

#### Gabapentin

**Fig. 5:**
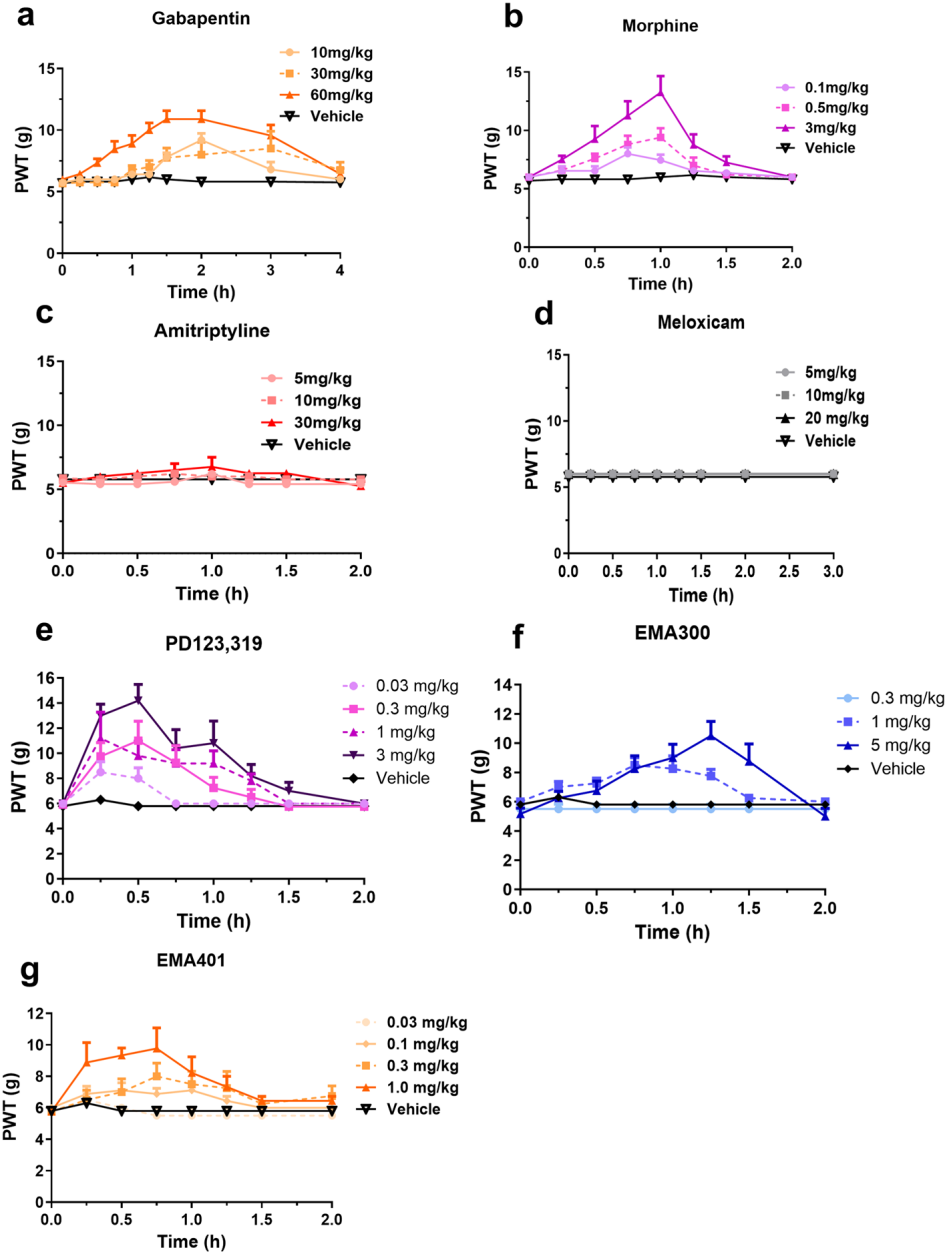
Single s.c. doses of **a** gabapentin (10–60 mg/kg) and **b** morphine (0.1–3 mg/kg) evoked dose-dependent anti-allodynia in the ipsilateral hindpaws of the VZV-induced rat model of neuropathic pain, whereas single i.p. doses of **c** amitriptyline (5–30 mg/kg) and **d** meloxicam (5–20 mg/kg) lacked efficacy in the dose ranges tested, similar to vehicle. Single doses of **e** PD123,319 (0.03–3 mg/kg i.v.), **f** EMA300 (0.3–5.0 mg/kg i.p.) and **g** EMA401 (0.03–1.0 mg/kg p.o.) evoked dose-dependent anti-allodynia in the VZV-induced rat model of neuropathic pain

Administration of single s.c. doses of gabapentin at 10–60 mg/kg evoked dose-dependent relief of ipsilateral hindpaw hypersensitivity in our VZV-rat model of neuropathic pain (Fig. 5a). The estimated ED_50_ (95% confidence interval, 95% CI) for the ipsilateral hindpaws was 39.3 (25.1–64.8) mg/kg and mean peak pain relief evoked by gabapentin was observed at ~ 1.5–3.0 h post-dosing (Fig. 5a). At the highest dose tested (60 mg/kg), mechanical allodynia in the ipsilateral hindpaws was fully alleviated at the time of peak effect; the corresponding mean duration of action was ~ 4 h. For the doses of s.c. gabapentin administered to the VZV-induced rat model of PHN, there were no discernible behavioural side effects evoked.

#### Morphine

In VZV-rats with fully developed mechanical allodynia in the ipsilateral hindpaws, administration of single s.c. doses of morphine at 0.1–3.0 mg/kg produced dose-dependent relief of ipsilateral hindpaw hypersensitivity (Fig. 5b). The ED_50_ was estimated at 0.74 mg/kg. Mean peak pain relief evoked by morphine was observed at 0.75–1.0 h post-dosing and at the highest dose tested (3.0 mg/kg), mechanical allodynia was fully alleviated at the time of peak effect with a corresponding mean duration of action of ~ 1.5 h (Fig. 5b). For the doses of s.c. morphine administered to VZV-rats herein, there were no discernible behavioural side effects produced.

#### Meloxicam and amitriptyline

In contrast with single bolus doses of gabapentin and morphine, single i.p. bolus doses of each of amitriptyline (5–30 mg/kg; Fig. 5c) and meloxicam (5–20 mg/kg; Fig. 5d) did not significantly alleviate mechanical hypersensitivity in the ipsilateral hindpaws, in a manner similar to vehicle.

#### PD123,319

Single i.v. doses of PD123,319 at 0.03–3.0 mg/kg evoked dose-dependent relief of mechanical allodynia in the ipsilateral hindpaws of VZV-rats (Fig. 5e). Mean peak anti-allodynia was observed at ~ 0.25–0.5 h post-dosing and the corresponding mean durations of action were 1.5–2 h. At the highest dose tested (3 mg/kg), mechanical allodynia was fully alleviated at the time of peak effect. The ED_50_ (95% CI) was 0.57 (0.04–1.7) mg/kg and there were no discernible behavioural side effects observed in the animals dosed with PD123,319.

#### EMA300

Administration of single i.p. doses of EMA300 at 0.3–5.0 mg/kg produced dose-dependent relief of ipsilateral hindpaw hypersensitivity in VZV-rats (Fig. 5f). Mean peak pain relief evoked by EMA300 was observed at ~ 1.0–1.5 h post-dosing. At the highest dose tested (5 mg/kg), mechanical allodynia was fully alleviated at the time of peak effect and the corresponding mean duration of action was ~ 2 h. The ED_50_ (95% CI) was 2.5 (1.0–3.7) mg/kg and there were no discernible behavioural side effects evoked in the dose range tested.

#### EMA401

Single oral doses of EMA401 at 0.03–1.0 mg/kg produced dose-dependent anti-allodynia in the ipsilateral hindpaws of VZV-rats (Fig. 5g). Mean peak pain relief was observed at ~ 0.75 h post-dosing. At the highest dose tested (1 mg/kg), mechanical allodynia was fully alleviated at the time of peak effect and the corresponding mean duration of action was ~ 2 h. The estimated ED_50_ (95% CI) was 0.41 (0.12–0.87) mg/kg and there were no discernible behavioural side effects evoked in the dose range tested.

## Discussion

Herein, we established a VZV-induced rat model of neuropathic pain by unilateral i.pl. injection of VZV-infected MRC-5 cells, recapitulating previous work by others in male Wistar rats infected with the Ellen strain of VZV propagated in CV-1 (African green monkey kidney fibroblast) cells (Dalziel et al. 2004), male Sprague–Dawley rats inoculated by the i.pl. route with the VZV-1755 and VZV-1587 strains propagated in MRC-5 cells or the VZV Parent of Oka strain propagated in MeWo cells (Kinchington and Goins 2011; Guedon et al. 2015). We also characterised this model pharmacologically using representative analgesic/adjuvant agents from five different pharmacological classes. Specifically, our pharmacological data show that both the analgesic adjuvant, gabapentin, and the strong opioid analgesic, morphine, evoked dose-dependent anti-allodynia in the ipsilateral hindpaws of the VZV-rat model of neuropathic pain. For gabapentin and morphine, the ED_50_ values of 39.9 and 0.74 mg/kg, respectively, extend previous work by others who showed single oral doses of gabapentin at 100 mg/kg (Garry et al. 2005), and twice-daily doses of gabapentin at 30 mg/kg i.p. or morphine at 2.5 mg/kg i.p. for 4 days, alleviated mechanical hypersensitivity in the hindpaws of similar VZV-rats (Hasnie et al. 2007). The anti-allodynic effect of gabapentin in VZV-rats is aligned with clinical studies showing that gabapentin and its structural analogue, pregabalin, alleviated neuropathic pain in patients with PHN (Rowbotham et al. 1998; Singh and Kennedy 2003; Dworkin et al. 2003; Menaldi et al. 2022; Cao et al. 2023).

Of novel interest herein, we show that single doses of the highly selective small molecule AT_2_ receptor antagonists, PD123,319 EMA300, and EMA401, evoked dose-dependent anti-allodynia in the ipsilateral hindpaws of VZV-rats. The potency of single i.v. doses of PD123,319 in VZV-rats is similar to that for single i.p. doses of PD123,319 for the relief of mechanical allodynia in the ipsilateral hindpaws of CCI-rats (3.2 mg/kg; Smith et al. 2013a) and in the hindpaws of rats with antiretroviral (dideoxycytidine; ddC) induced toxic neuropathy (3.2 mg/kg) (Smith et al. 2014). The time to peak effect (*T*_max_) for i.v. PD123,319 in VZV-rats is shorter at 0.25–0.5 h compared with 0.5–0.75 h for i.p. PD123,319 in CCI-rats (Smith et al. 2013a) and 1.5 h in ddC-rats (Smith et al. 2014). The mean durations of action for single doses of PD123,319 in VZV-rats, CCI-rats and ddC-rats are similar at ~ 1.5–2 h. The potency of i.v. PD123,319 in VZV-rats is two–threefold higher than the potency in a rat model of prostate cancer induced bone pain, a type of chronic pain underpinned by both inflammatory and neuropathic components (Muralidharan et al. 2014). However, the time to peak pain relief evoked by i.v. PD123,319 was similar between these two rodent models of persistent pain.

In other work using a mouse model of gout induced by intraarticular injection of monosodium urate crystals into the ankle joint, pretreatment with single oral or intraarticular doses of PD123,319, prevented the development of mechanical hypersensitivity in the ipsilateral hindpaws (Vieira et al. 2022). The AT_2_ receptor was confirmed as the target as PD123,319 pain relief was abolished in mutant *Agtr2*^tm1a^ mice that had the AT_2_ receptor effectively knocked out (Vieira et al. 2022). In other work in mice, i.pl. Ang II (10–300 pmol) evoked a prolonged period (24 h) of mechanical hypersensitivity in the ipsilateral hindpaws that was abolished in AT_2_ but not AT_1_ receptor knockout (KO) mice, further emphasising the pro-nociceptive effects of Ang II signalling via the AT_2_ receptor (Shepherd et al. 2018). As i.pl. (but not intrathecal) injection of PD123,319 at 10 pmol in mice attenuated i.pl. Ang II-evoked mechanical hypersensitivity in the ipsilateral hindpaws, i.pl. PD123,319 evoked its anti-hyperalgesic effect via a peripheral mechanism (Shepherd et al. 2018). This i.pl. Ang II-evoked hindpaw hypersensitivity was associated with increased production of reactive oxygen species (ROS) at the injection site which was attenuated by co-administration of PD123,319 or the antioxidant *N*-acetylcysteine (Shepherd et al. 2018). As macrophages are prodigious producers of ROS, chemogenetic ablation of macrophages was used to further assess a role for ROS in mediating Ang II-induced hindpaw hypersensitivity (Shepherd et al. 2018). This notion was supported as macrophage ablation in nerve-injured mice abolished mechanical and cold hypersensitivity and reconstitution of wild-type mice with AT_2_ receptor KO donor bone marrow, attenuated nerve injury-induced hypersensitivity significantly (Shepherd et al. 2018).

Comparison of the ED_50_ for i.p. EMA300 in VZV-rats (2.9 mg/kg) with that for CCI-rats (0.8 mg/kg) (Smith et al. 2013a) shows that EMA300 is ~ 3.6-fold less potent in the VZV-induced rat model of neuropathic pain compared with the CCI-model. The mean times to peak effect after single i.p. doses of EMA300 in VZV- and CCI-rats were 0.75–1.25 h and 0.5–0.75 h, respectively (Smith et al. 2013a), but the corresponding mean durations of action were ~ 2 h for both models. Importantly, the anti-allodynic efficacy of EMA300 was abolished in CCI-mice null for the AT_2_ receptor with intermediate effects in the hemizygotes, confirming that EMA300 pain relief was mediated by the AT_2_ receptor (Smith et al. 2013b). At the time of peak EMA300 pain relief in CCI-rats, otherwise augmented Ang II levels signalling via the AT_2_ receptor in the ipsilateral lumbar dorsal root ganglia (DRGs) of CCI-rats were attenuated to the corresponding levels in the sham-operated animals, which in turn inhibited activation of p38 MAPK and p44/p42 MAPK (Smith et al. 2013b). In work by others using cultured DRG neurons, Ang II signalling via the AT_2_ receptor promoted neurite outgrowth and augmented capsaicin-induced cultured neuronal hyperexcitability (Gendron et al. 1999; Stroth et al. 2000; Plouffe et al. 2006; Chakrabarty et al. 2008; Anand et al. 2013), a pathobiological hallmark of neuropathic pain (Basbaum et al. 2009).

The anti-allodynic potency of single oral doses of EMA401 in VZV-rats herein (ED_50_ ~ 1.0 mg/kg) was lower than that for i.p. EMA400 (racemic mixture of *S*- and *R*-enantiomers), in CCI-rats (ED_50_ ~ 0.01 mg/kg) (Smith et al. 2013a). As the oral bioavailability of EMA401 relative to i.v. EMA401 is 33% (Smith et al. 2013a), the lower potency of oral EMA401 in VZV-rats compared with i.p. EMA400 in CCI-rats is likely underpinned primarily by between-model differences in the pathobiology of these two neuropathic pain conditions. The mean times to peak effect for oral EMA401 in VZV-rats and i.p. EMA400 in CCI-rats (Smith et al. 2013a) were 0.5–0.75 h. The corresponding mean durations of action were ~ 3 h. The lack of discernible CNS side effects following administration of single oral doses of EMA401 in VZV-rats herein is supported by the fact that EMA401 is peripherally restricted, as ^14^C was undetectable in the central nervous system of rats administered ^14^C-labelled EMA401 when assessed using whole-body autoradiography (Koyama et al. 2018; Guo et al. 2021). The latter finding also explains the lack of CNS side effects in early phase clinical trials of EMA401 in healthy volunteers and patients with PHN (Rice et al. 2014; Rice et al. 2021).

The dose-dependent anti-allodynia evoked by several small molecule AT_2_ receptor antagonists in a rat model of VZV-induced neuropathic pain herein provides additional data on the pharmacological characterisation of this model. Importantly, the anti-allodynic efficacy of oral EMA401 in VZV-rats re-capitulated the successful analgesic efficacy observed in a randomised, double-blind, placebo-controlled Phase 2a clinical trial of oral EMA401 at 100 mg administered twice-daily for 4 weeks to 183 patients with PHN (Rice et al. 2014). The translation of promising preclinical findings for a novel investigational agent for the relief of neuropathic pain from the Smith laboratory to a successful Phase 2 clinical trial in patients with PHN is significant, as most new molecules modulating other novel “pain targets” in studies using rodent neuropathic pain models over the past decade, have failed to translate to a successful Phase 2 clinical trial.

Regarding the mechanism through which the anti-allodynic effects of small molecule AT_2_ receptor antagonists are mediated in the VZV-rat model of neuropathic pain, it is likely that a similar neuroimmune mechanism to that described for other neuropathic pain models (Shepherd et al. 2023) is responsible, but this remains for future investigation.

Based upon the hypothesis that the unexpected hepatotoxicity of oral EMA401 given once-daily for 39 weeks to cynomolgus monkeys in a toxicity study undertaken concurrently with Phase 2b clinical trials (Rice et al. 2021) is molecule-specific, a plausible mechanism involves formation of an acyl glucuronide metabolite which in turn may form covalent adducts with proteins that then act as haptens to the immune system. Such a mechanism for hepatotoxicity has been reported for some nonsteroidal anti-inflammatory drugs that also contain carboxylic acid functional groups in their chemical structures resulting in withdrawal from the market (Higton et al. 2021). Hence, work aimed at discovery of novel AT_2_ receptor antagonists without a carboxylic acid functional group in their chemical structure to avoid formation of potentially hepatotoxic acyl glucuronide metabolites is worthy of future investigation.

Limitations of the present work that need to be addressed in future work beyond the scope of that described herein are assessment of the potential for tolerance development and abuse liability with chronic dosing of AT_2_ receptor antagonists in rodents, although such issues were not reported in phase 2 clinical trials of oral EMA401 undertaken to date (Rice et al. 2014, 2021). In terms of translatability, our present findings show that the VZV-induced rat model of neuropathic pain is suitable for pain relief efficacy assessment of AT_2_ receptor antagonists from drug discovery recapitulating the analgesic efficacy of a Phase 2a clinical trial of oral EMA401 in patients with PHN (Rice et al. 2014). Particular attention to discovery of novel AT_2_ receptor antagonists that do not have a carboxylic acid functional group in their chemical structures whilst retaining pain relief efficacy in the VZV-rat model of neuropathic pain described herein has value for identifying AT_2_ receptor antagonists worthy of progressing to hepatotoxicity testing following prolonged chronic dosing in cynomolgus monkeys.

## Conclusion

We pharmacologically characterised a VZV-induced rat model of neuropathic pain using representative compounds of four clinically used classes of analgesic/adjuvant agents including gabapentin, amitriptyline, morphine and meloxicam. In addition, we further characterised this model using several highly selective, small molecule AT_2_ receptor antagonists, viz. PD123,319, EMA300 and EMA401, showing that they evoked dose-dependent anti-allodynia in the ipsilateral hindpaws of the VZV-rat model recapitulating a successful Phase 2a clinical trial of oral EMA401 in patients with PHN. Thus, our findings show that the VZV-induced rat model of neuropathic pain has utility for in vivo pharmacological assessment of novel AT_2_ receptor antagonists from analgesic drug discovery programs.

## Supplementary Information

Below is the link to the electronic supplementary material.

Supplementary file1 (DOCX 1610 KB)

## Data Availability

Data is available upon request from the Authors.
