# Supplementary material for: The angiotensin II type 2 receptor antagonists, PD123,319 ((S-( +)-1-[(4-(dimethylamino)-3-methylphenyl)methyl]-5-(diphenylacetyl)-4,5,6,7-tetrahydro-1H-imidazo[4,5-c]pyridine-6-carboxylic acid), EMA300 (5-(2,2-diphenylacetyl)-4-[(4-methoxy-3-methylphenyl)methyl]-1,4,6,7-tetrahydroimidazo[4,5-c]pyridine-6-carboxylic acid) and EMA401 ((3S)-5-(benzyloxy)-2-(2,2-diphenylacetyl)-6-methoxy-1,2,3,4-tetrahydroisoquinoline-3-carboxylic acid), evoke pain relief in a varicella zoster virus-induced rat model of neuropathic pain
Source: Inflammopharmacology. 2025 Feb 20;33(3):1337–48. doi: 10.1007/s10787-025-01650-z (PMC11913958; doi:10.1007/s10787-025-01650-z)
Supplement: Supplementary file 1 — Supplementary file1 (DOCX 1610 KB) [file 10787_2025_1650_MOESM1_ESM.docx]

**Appendix A: Supplementary Information**

**The angiotensin II type 2 receptor antagonists, PD123,319 ((S-(+)-1-[(4-(dimethylamino)-3-methylphenyl)methyl]-5-(diphenylacetyl)-4,5,6,7-tetrahydro-1H-imidazo[4,5-c]pyridine-6-carboxylic acid), EMA300 (5-(2,2-diphenylacetyl)-4-[(4-methoxy-3-methylphenyl)methyl]-1,4,6,7-tetrahydroimidazo[4,5-c]pyridine-6-carboxylic acid) and EMA401 ((3S)-5-(benzyloxy)-2-(2,2-diphenylacetyl)-6-methoxy-1,2,3,4-tetrahydroisoquinoline-3-carboxylic acid) evoke pain relief in a varicellar zoster virus-induced rat model of neuropathic pain**

Das V^1^, Lam AL^1^, Smith MT^1^*

School of Biomedical Sciences, Faculty of Medicine, The University of Queensland, St Lucia Campus, Brisbane, Queensland, Australia. 4072

*Corresponding author

Em. Professor Maree T Smith

Director, Centre for Integrated Preclinical Drug Development (CIPDD)

School of Biomedical Sciences

The University of Queensland

St Lucia Campus.

Brisbane

Queensland, 4072.

Australia

In this Supplementary Information, the *in vitro* methods used to characterise the Ellen strain of Varicellar Zoster Virus (VZV) propagated in MRC-5 cells are described. We used VZV-infected MRC-5 cells to establish an *in vivo* rat model of VZV-induced neuropathic pain as described in the accompanying main paper.

**Supplementary Methods**

**Immunocytochemical detection of VZV-immediate early IE-62 protein**

Mouse monoclonal anti-VZV IE-62 antibody (MAB8616) was from Millipore (Melbourne, VIC, Australia). Normal goat serum and Alexa Fluor® 546 Goat Anti-Mouse IgG (H+L) were from Invitrogen (Melbourne, VIC, Australia). Hydrogen peroxide 30% w/v was from UNIVAR (Sydney, NSW, Australia), and Dako fluorescent mounting media was from Dako (Melbourne, VIC, Australia). Fish skin gelatine and Triton X-100 were from Sigma-Aldrich (Sydney, NSW, Australia). Paraformaldehyde (PFA) was from PST ProSciTech (Brisbane, QLD, Australia).

**RT-PCR detection of immediate early IE-62 gene**

Chloroform was from Sigma-Aldrich (Sydney, NSW, Australia). UltraPure™ DNase/RNase-Free Distilled Water was purchased from Invitrogen (Melbourne, VIC, Australia). IE-62 primer & designated open reading frame (ORF), (VZV-ORF62F (5’- TCT TGT CGA GGA GGC TTC TG’- 3’) and VZV-ORF62R (5’- TGT GTG TCC ACC GGA TGA T-3’)) and IE-62 probe (VZV-ORF62probe (5’- TCT CGA CTG GCT GGG ACT TGC G – 3’)) (TaqMan TAMRA), TaqMan® Gene Expression Master Mix, high-capacity RNA-to-cDNA kit, and Pre-Developed TaqMan® assay reagent Human GAPDH, RNAqueous®-4PCR Kit were from Applied Biosystems (Melbourne, VIC, Australia).

**Propagation of Varicella Zoster Virus**

The MRC-5 cell line (CCL-171^TM^) derived from human fetal lung fibroblasts), purchased from American Type Culture Collection (ATCC; Manassas, VA, USA), was evaluated for its suitability for propagating VZV in cell culture. MRC-5 cells were cultured at 37^o^C with 5% CO_2_ in a humidified incubator in Minimum essential medium (MEM) containing 10% foetal bovine serum (FBS), glutamax (2 mM), sodium pyruvate (1 mM) and 100U penicillin-streptomycin (all from Invitrogen, Melbourne, Vic, Australia). MRC-5 cells were grown and passaged multiple times in T-75 flasks to about 80% confluence, followed by cell count using trypan blue exclusion staining.

MRC-5 cells were inoculated with the Ellen strain of VZV (VR-1367^TM^) and these infected cells were incubated at 37^o^C in a humidified 5% CO_2_ cell culture incubator for approximately 48 hours. Propagation of VZV was carried out until ~75-80% of cells displayed a cytopathic effect (CPE) characterized by morphological changes in cells that included cell swelling or shrinkage, detachment from the growth surface and/or cell death. Further propagation of the VZV strain was performed by trypsinization of the VZV-infected cells and subsequent inoculation onto uninfected host cells at a ratio range of 1:4 to 1:10.

Cryopreserved stocks of MRC-5 cells infected with the Ellen strain of VZV were prepared by trypsinization of VZV-infected MRC5 cells in a T-75 flask followed by centrifugation at 130g for 5 minutes at 4°C, and the resulting pellets were resuspended in a 2ml mixture comprising MEM medium supplemented with 10% FBS and 10% DMSO (dimethylsulfoxide). The cell suspensions were transferred into cryovial tubes (1ml/vial) and stored in liquid nitrogen until use.

**Assessment of virus infectivity**

To assess the *in vitro* infectivity of the Ellen strain of VZV, virus infectivity tests were carried out in 96-well plates. Aliquots (100µL) of MRC-5 cell suspensions (2x10^5^cells/ml) were seeded into each well of a 96 well plate and grown at 37°C in 5% CO_2_ in a humidified incubator for 24-48 hours until ~80% confluent. The Ellen strain was propagated in MRC-5 cells as described above, and harvested by trypsinization when the infected cells displayed 75-80% CPE. The VZV-infected cells were resuspended in growth medium to a final stock concentration of 2x10^5^ cell/ml. 10-fold serial dilutions of the 2x10^5^ cells/ml VZV-infected MRC-5 cells were prepared and added (100µL) to the 96-well plates containing MRC-5 cells. Cells were incubated for 7 days at 37°C in 5% CO_2_ in a humidified incubator. The infectivity titre of the Ellen strain was assessed semi-quantitatively by staining the cells in the 96-well plates with crystal violet (0.5% w/v in 70% methanol).

**S2.3 qRT-PCR analysis of IE-62 mRNA expression**

MRC-5 cells were propagated to ~75% confluence in T-75 flasks prior to inoculation with Ellen-infected MRC-5 cells in T-75 flasks. VZV-propagation was carried out at 37^o^C in a humidified 5% CO_2_ incubator for approximately 48 hours. RNA (ribonucleic acid) was isolated from non-infected MRC-5 cells as well as MRC-5 cells infected with the Ellen strain of VZV using RNAqueous®-4PCR Kits according to the manufacturer’s instructions. Isolated RNA was converted to cDNA (complementary DNA) using a high-capacity RNA-to-cDNA kit according to the manufacturer’s instructions and stored at -80^o^C. Master mixes for human GAPDH (glyceraldehyde 3-phosphate dehydrogenase) (5µl 2X TaqMan® Gene Expression Master Mix, 0.5µl 20X human GAPDH primer and probe mixture, and 2.5µl UltraPure™ DNase/RNase-Free Distilled Water for each reaction) and IE-62 (5µl 2X TaqMan® Gene Expression Master Mix, 0.18µl VZV-ORF62F, 0.18µl VZV-ORF62R, 0.25µl VZV-ORF62 probe and 2.39µl UltraPure™ DNase/RNase-Free Distilled Water for each reaction) were prepared.

As quantitative real-time PCR reaction is affected to a large extent by the amplification efficiency of the PCR (polymerase chain reaction), the amplification efficiency of IE62 and human GAPDH were determined using RNA isolated from MRC-5 cells infected with the Ellen strain of VZV. Briefly, the cDNA samples generated from the VZV-infected MRC-5 cells were diluted in fivefold serial dilutions with UltraPure™ DNase/RNase-Free Distilled Water (1/5 to 1/3125). qRT-PCR (reverse transcription quantitative PCR) reactions were performed in triplicate wells in a 384-well plate with each well containing 8 µL of one of the master mixes (IE62 or human GAPDH) and 2 µL of cDNA. Assays were carried out using the following PCR conditions: 50^o^C for 2 minutes (1X), 95^o^C for 10 minutes (1X) and 95^o^C for 15 seconds and 60^o^C for 1 minute (45X) on an ABI PRISM® 7900HT Sequence Detection System (Applied Biosystems). A plot of Ct vs. log cDNA concentrations for the Ellen strain was constructed to generate a linear regression slope, to which amplification efficiency for the IE-62 and human GAPDH probes were determined by the following formula:

Amplification efficiency (E) = 10^-1/slope^

Relative quantification of IE62 expression to human GAPDH for each cell line infected with the Ellen strain of VZV was calculated with the formula below:

Target gene/reference gene = (E_ref_)^C^_t_^ref^/(E_target_)^C^_t_^target^

where target gene = IE-62, reference gene = GAPDH, E = amplification efficiency, C_t_ = threshold cycle

**Detection of IE-62 in VZV-infected cells by immunocytochemistry (ICC)**

Non-infected MRC-5 cells as well as MRC-5 cells infected with the Ellen VZV strain (~75-80% CPE) were grown on cover slips, fixed with 4% PFA and washed three times with Dulbecco’s Phosphate Buffered Saline (DPBS). Fixed cells were blocked with 10% normal goat serum (NGS) for 1 hour at room temperature and incubated at 4^o^C overnight with mouse monoclonal anti-VZV IE-62 antibody (1:250 dilution) under humidified conditions. The cells were washed three times with DPBS and incubated with Alexa Fluor® 546 Goat Anti-Mouse IgG (H+L) (1:10,000 dilution) for 1 hour at room temperature. The cells were washed with DPBS, air dried under humidified conditions and coverslips were placed on top of glass slides. Images were captured using the Axio Vision image analysis and processing software (Zeiss, Axiovision Rel.v4.8 software).

**Supplementary Results**

**Cell culture and propagation of virus**

Figure S1 shows images of (**a**) propagated uninfected MRC-5 cells and (**b**) propagated MRC-5 cells infected with the Ellen strain of VZV showing CPE, respectively.

**Assessment of virus infectivity**

Viral infectivity of the Ellen strain of VZV was assessed in MRC-5 cells by addition of 10-fold serial dilutions of the Ellen strain into a 96-well plate containing a monolayer of MRC-5 cells. The viral infectivity titre was determined as the higest dilution that resulted in CPE in any of the replicate wells. The infectivity titre for the Ellen strain of VZV propagated in MRC-5 cells was estimated at 10^-4^ µl.

**IE-62 mRNA expression of VZV strains in MRC-5 cells**

The VZV genome comprises 125kbp that encode approximately 71 open reading frames (ORFs) encoding 68 proteins (Khalil et al., 2008). IE-62 is one of the major transcriptional activator proteins encoded by VZV that locates to the nucleus when expressed in transfected cells (Kinchington et al., 2000). As quantitative real-time PCR reactions can be markedly affected by the amplification efficiency of a particular target gene within a PCR assay, the amplification efficiencies of IE62 and GAPDH were determined using a 5-fold dilution series of cDNAs obtained from MRC-5 cells that were infected with the Ellen strain of VZV.

Ampli­fication efficiency of the reaction is an important element when performing relative quantitation by RT-PCR with an ideal amplification efficiency of 1, where the PCR product doubles during every cycle within the exponential phase of the reaction. The amplification efficiency for IE-62 for MRC-5 cells infected with the Ellen strain of VZV was 2.06. Amplification efficiencies for human GAPDH for non-infected MRC-5 cells and MRC-5 cells infected with the Ellen strain were 2.24 and 2.08 respectively. Using the amplification efficiency values, the calculated relative expression of IE-62:GAPDH for non-infected MRC-5 cells and MRC-5 cells infected with the Ellen strain of VZV were 0.0 and 4.06 respectively (Table S1).

**Detection of IE-62 protein in VZV-infected cells**

The presence of VZV in MRC-5 cells was further confirmed by detection of IE-62 using immunocytochemistry (ICC). Optimal conditions for detection of IE-62 protein in Ellen-infected MRC-5 cells by ICC were confirmed using a 1:250 dilution of mouse monoclonal anti-VZV IE-62 antibody and a 1:10,000 dilution of Alexa Fluor® 546 Goat Anti-Mouse IgG (H+L) antibody (Figure S2).


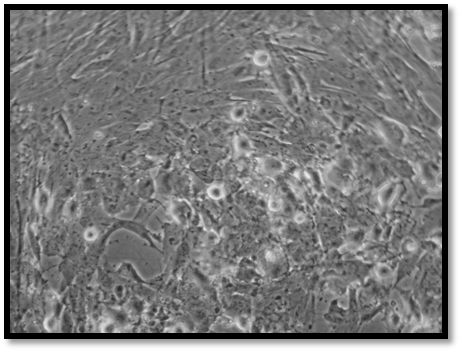


**b**


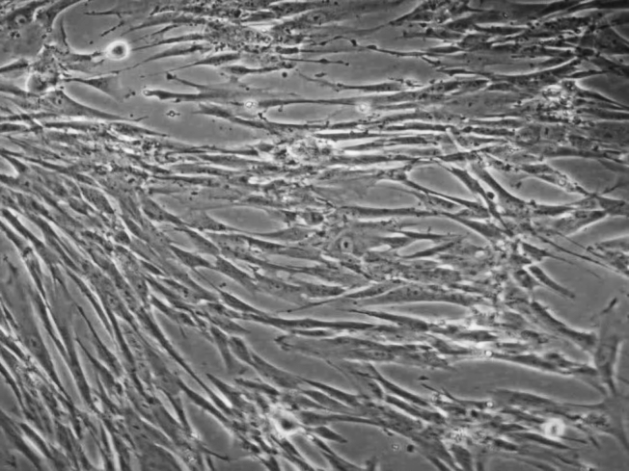


**a**

**A**

**A**

**Non-infected MRC-5 cells**

**C**

**C**

**MRC-5 cells infected with the Ellen strain of VZV**

**Figure S1**. Propagation of the Ellen Strain of VZV in cultured MRC-5 cells. (**a**) non-infected MRC-5 cells; (**b**) MRC-5 cells infected with the Ellen strain of VZV showing CPE.

**Table S1:** Amplification efficiency and relative quantification of IE-62 and GAPDH in uninfected MRC-5 cells and MRC-5 cells infected with the Ellen strain of VZV

|  | **IE-62** | | | **GAPDH** | | |  |
| --- | --- | --- | --- | --- | --- | --- | --- |
|  | **Slope** | **Efficiency** | **Average C_t_ value** | **Slope** | **Efficiency** | **Average C_t_ value** | **Relative quantification** |
| MRC-5 cells | ND | ND | ND | -2.85 | 2.24 | 16.37 | 0.0000 |
| Ellen-infected MRC-5 cells | -3.18 | 2.06 | 14.67 | -3.13 | 2.08 | 16.39 | 4.06 |

ND= not determined


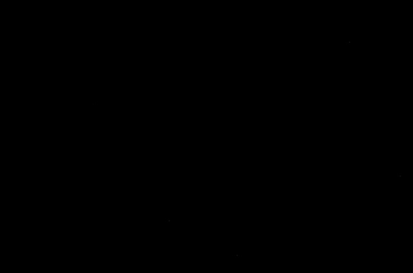


**a**

**b**

**
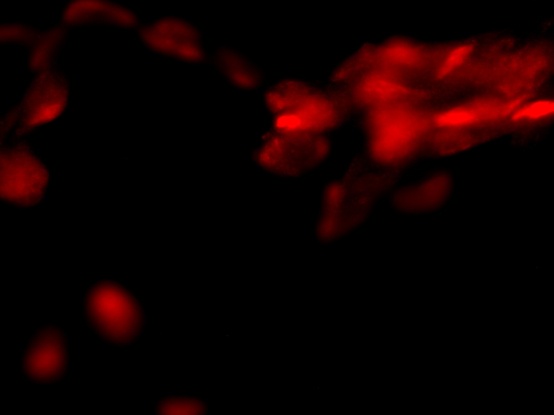
**

**Figure S2**. Detection of VZV IE-62 protein by immunocytochemistry (ICC). (**a**) Absence of IE-62 expression in non-infected MRC-5 cells. IE-62 expression in MRC-5 cells infected with (**b**) the Ellen strain of VZV.

**References**

1. Khalil, M. I., Hay, J., & Ruyechan, W. T. (2008). Cellular transcription factors Sp1 and Sp3 suppress varicella-zoster virus origin-dependent DNA replication. *J Virol, 82*(23), 11723-11733. doi:10.1128/JVI.01322-08
2. Kinchington, P. R., Fite, K., & Turse, S. E. (2000). Nuclear accumulation of IE62, the varicella-zoster virus (VZV) major transcriptional regulatory protein, is inhibited by phosphorylation mediated by the VZV open reading frame 66 protein kinase. *J Virol, 74*(5), 2265-2277. doi:10.1128/jvi.74.5.2265-2277.2000
